# Repetitive transcranial magnetic stimulation for insomnia in patients with autism spectrum disorder: Study protocol for a randomized, double-blind, and sham-controlled clinical trial

**DOI:** 10.3389/fpsyt.2022.977341

**Published:** 2022-09-28

**Authors:** Jian Jiao, Lu Tan, Ye Zhang, Taomei Li, Xiangdong Tang

**Affiliations:** Sleep Medicine Center, Mental Health Center, Translational Neuroscience Center, West China Hospital, Sichuan University, Chengdu, China

**Keywords:** autism spectrum disorder, repetitive transcranial magnetic stimulation, clinical trial, insomnia, neuromodulation

## Abstract

**Background:**

Insomnia is the most common comorbidity in children with autism spectrum disorder (ASD) and seriously affects their rehabilitation and prognosis. Thus, an intervention targeting insomnia in ASD seems warranted. Repetitive transcranial magnetic stimulation (rTMS), a potentially effective treatment for improving sleep quality and optimizing sleep structure, has already been demonstrated to alleviate insomnia symptoms and sleep disturbance in different neurological and neuropsychiatric conditions. This trial aims to investigate the effects of rTMS on insomnia in patients with ASD.

**Method:**

This study is designed to be a double-blind, randomized, and sham-controlled trial with a target sample size of 30 participants (aged 3–13 years) diagnosed with ASD comorbid with insomnia. The intervention phase will comprise 20 sessions of rTMS or sham rTMS applied over the right dorsolateral prefrontal cortex (DLPFC) within four consecutive weeks. The effect of rTMS on insomnia and other symptoms of ASD will be investigated through home-PSG (two consecutive overnights), sleep diary, CSHQ, CARS, ABC, SRS, RBS-R, and metabolomics analysis at baseline and posttreatment. A follow-up assessment 1 month after the intervention will examine the long-term effects.

**Discussion:**

The results of this study may address an important knowledge gap and may provide evidence for the use of rTMS to treat insomnia in ASD. Furthermore, it will elucidate the potential mechanism and link between sleep disorders and clinical symptoms.

**Clinical trial registration:**

The study is ongoing and has been registered at the Chinese Clinical Trial Registry (ChiCTR2100049266) on 28/07/2021.

## Introduction

Autism spectrum disorder (ASD) is a group of neurodevelopmental disorders with significant clinical heterogeneity characterized by social communication deficits and restricted, repetitive sensory–motor behaviors (1, 2). The prevalence of ASD has increased to 1/59, and over 70% of individuals with autism have developmental problems (intellectual disability, etc.), general medical issues (sleep disturbances, gastrointestinal problems, etc.), psychiatric conditions (anxiety and depression, etc.), or personality disorders (schizoid personality disorder, etc.) to varying degrees (2, 3).

Among these comorbidities, sleep disturbances, especially insomnia, are the most common conditions, and their prevalence in ASD is 40–80%, which is two to three times higher than that in the general population (4–6). Previous studies have found a bidirectional association between ASD and sleep disturbances (7). Furthermore, like the core symptoms of autism, insomnia may persist throughout life and can not only predict the severity of core symptoms and related daily behavior problems, such as tantrums and aggression, but also affect the health and wellbeing of parents (6, 8). Given the significant adverse impacts, treating insomnia has therefore been a primary therapeutic goal.

Over the years, lifestyle modifications, behavioral interventions, and pharmacological therapies have been adopted for treating sleep disturbances in ASD (9, 10). Among them, cognitive behavioral therapy for insomnia (CBT-I), as a first-line treatment to manage chronic insomnia, has also been used to treat sleep disturbances in children with ASD. One study of school-aged children with high-functioning ASD demonstrated that CBT-I was a feasible and promising treatment to improve the sleep and function of children and parents (11). Nevertheless, poor treatment responses (25%) were reported in ASD with parent-directed behavioral sleep interventions due to numerous ASD children having atypical cognitive profiles, such as impaired social cognition and atypical perceptual and information processing, which led to poor cooperation during the treatment (12, 13). As a stepwise approach to sleep disturbances, pharmacotherapy is often provided when behavioral intervention fails. Nevertheless, there are no medications with regulatory approval by the Food and Drug Administration (FDA) for the treatment of insomnia in ASD (9). Considering the severity of sleep problems, drugs including antidepressants, antipsychotics, benzodiazepines, and melatonin have been used off-label in the clinic with some potential side effects (14, 15). From the perspective of the parents, behavioral and nonpharmacological interventions are generally the first choice over equally effective medication (9). However, existing treatments are less effective. Therefore, identifying a safe and effective nonpharmacological treatment for insomnia in children with ASD is an urgent matter.

Repetitive transcranial magnetic stimulation (rTMS) is a noninvasive therapy that produces a magnetic field and introduces excitatory or inhibitory cortical excitability in the brain below the magnetic coil by modulating different stimulus frequencies (16). rTMS has been used to treat major depression and treatment-resistant depression (17). Recently, numerous studies have adopted high- or low-frequency rTMS protocols with positive results in in hemiparesis due to pediatric stroke, mild traumatic brain injury, and tics/Tourette syndrome (18, 19). However, despite the benefits demonstrated for adult migraine, rTMS has been insufficiently studied for migraine in children. In addition, evidence for rTMS in childhood epilepsy and ADHD remains mixed (19). In ASD, the results confirmed that by stimulating different brain regions, different clinical symptoms were correspondingly improved: stimulating the dorsal lateral prefrontal cortex (DLPFC) can improve irritability, repetitive behaviors, and executive functioning; stimulating primary motor cortices can improve motor behavior; stimulating the medial prefrontal cortex can improve mentalizing; and stimulating the premotor cortex can improve speech production and eye-hand coordination (20). Regarding the adverse effects of TMS in children, studies found that the overall rate of the adverse effects, including headache, facial discomfort, irritability, pain at the application site, headedness, and dizziness, is 25% (18, 19, 21). However, all these adverse effects are mild and transient and can be resolved after rest or medication (21).

Moreover, meta-analysis and systematic reviews have indicated that low-frequency rTMS applied to the dorsolateral prefrontal cortex is safe and has the potential to alleviate insomnia symptoms and sleep disturbance in different neurological and neuropsychiatric conditions (22–24). Studies have found that rTMS not only improves sleep quality and optimizes sleep structure but also maintains therapeutic efficacy over pharmacological treatments and cognitive behavioral therapy and has emerged as a promising tool for treating sleep disturbances (22). Moreover, after 10 daily sessions of rTMS treatment in patients with primary insomnia, the concentrations of serum GABA and brain-derived neurotrophic factor (BDNF) were significantly higher than baseline (25). As we already know, GABA and BDNF both have a close relationship with sleep regulation (26–28). The change in metabolomics may be the potential mechanism of rTMS treatment in alleviating insomnia (28).

However, the implications of rTMS for insomnia in ASD patients have not been fully established. Only one study indicated that rTMS can alleviate sleep disturbances in ASD, which were assessed by the Children's Sleep Habits Questionnaire (CSHQ) (29). No randomized, double-blind, controlled studies were conducted to evaluate the efficacy of rTMS for insomnia through objective assessments such as PSG or actigraphy. Therefore, we aim to conduct a randomized, double-blind, sham-controlled trial to explore the effect of rTMS on insomnia with an objective assessment of ASD. Additional objectives include explorations of the effects of rTMS on ASD symptoms and the association between insomnia and ASD symptoms. We hypothesize that 1) rTMS applied to the right dorsolateral prefrontal cortex (DLPFC) can alleviate sleep quality and ASD symptoms and 2) metabolic changes may play a mediating role in sleep quality and ASD symptoms.

## Methods and analysis

### Study design and settings

**Figure 1:**
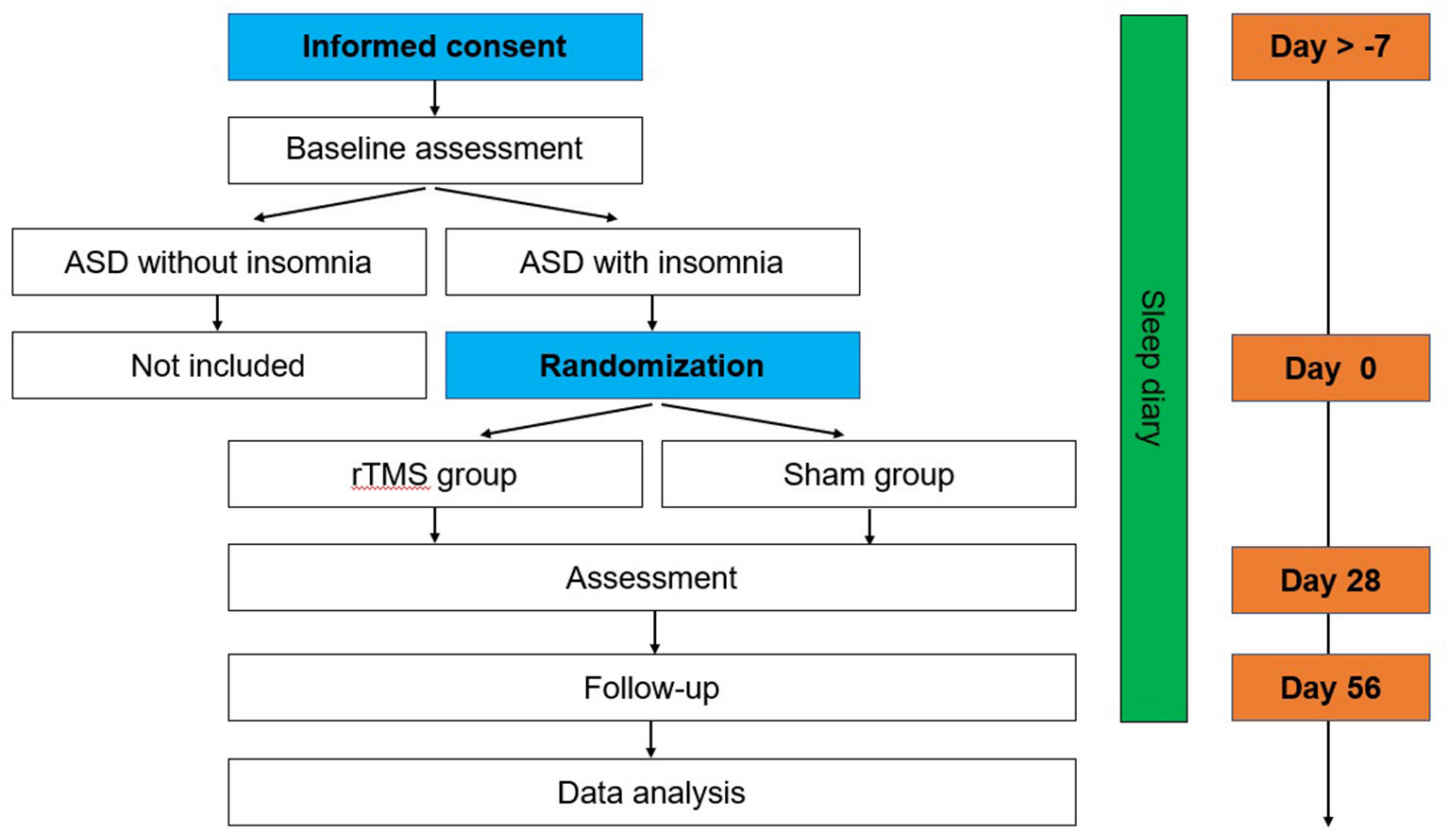
Time schedule of screening, interventions, assessments and visits of participants.

This is a double-blind, sham-controlled pilot trial to examine the efficacy of rTMS on insomnia in ASD. Participants will be randomized to the rTMS group or sham rTMS group for 4 weeks (5 days/week). Assessments of the primary and secondary outcomes will be performed at baseline, after the 4-week treatment, and at the 4-week follow-up with rTMS or sham treatment (Figure 1). The protocol was approved by the Biomedical Research Ethics Committee West China Hospital of Sichuan University and is registered at the Chinese Clinical Trial Registry (ChiCTR2100049266).

### Participants

Participants will be recruited from the special education schools, nursery and primary schools, the second outpatient department of the West China Mental Health Center of Sichuan University, and online. Measurements and interventions will be carried out at the second outpatient department of the West China Mental Health Center of Sichuan University.

### Sample size

Previous studies in patients with insomnia have shown that rTMS treatment can decrease sleep onset latency by at least 17.88–44.8 min (30, 31). According to previous studies, the minimally important difference of the primary outcome (sleep onset latency) is assumed to be 10 min (SD, 8.4 min) (32). To detect this difference with a two-sided significance level of 0.05 and a power of 80%, and account for the drop rate of 10% according to existing rTMS studies in autism (33, 34), a total of 30 participants will be included (PASS, version 15).

### Inclusion/exclusion criteria

Participants eligible for the trial must comply with all of the following:

1) A preliminary clinical diagnosis using DSM-5 in the Mental Health Center by a child psychiatrist will be confirmed by two well-trained child psychiatrists using the Autism Diagnostic Interview-Revised (ADI-R) and Autism Diagnostic Observational Schedule (ADOS-2) (35, 36).
2) Age between 3 and 13 years.
3) Patients with ASD must meet the following diagnostic criteria for insomnia in the International Classification of Sleep Disorders–Third Edition (ICSD-3) (duration > 3 months: difficulty sleeping (sleep latency > 20 min or wake-up time > 20 min after falling asleep) at least 3 nights per week, confirmed by sleep diary (at least 7 days) and portable home-PSG (2 consecutive nights, based mainly on the second-day monitoring data) (37).
4) Not taking any drugs that affect sleep or no change in the drug dose and not taking new drugs during the trial period.
5) Two living parents who are able to cooperate in signing the informed consent form and documents pertaining to related scales.

Participants will be excluded from the trial if they comply with one of the following:

1) Those who have a history of epilepsy or severe head trauma, who have medical device implants (such as pacemakers, etc.).
2) Participants cannot cooperate.
3) Severe neurologic or psychiatric disorders or medical conditions (e.g., skull defect and craniotomy) or other disorders likely to have an impact on sleep.

Participants who cannot tolerate the side effects (headache, etc.), develop any adverse events (seizure, etc.) during the treatment, or miss more than two rTMS treatment sessions in a row, or miss more than four sessions in total, will be withdrawn from this study.

### Randomization and blinding

For participant allocation, a computer-generated random list will be used, which is based on the permutation block method with a random number generator and allocated at a ratio of 1: 1. A research assistant concealed the allocation sequence using sealed, opaque envelopes that contain the treatment information (A or B) before officially entering the study. After completing the consent form and baseline measures, the corresponding envelope for a given participant ID will be given to the rTMS therapist prior to the first rTMS session. The rTMS therapist is the only person who determines the treatment method (rTMS or sham treatment) for A or B. Once the treatment information is decided, no change will be accepted. Others, such as study investigators, parents, patients and statistical analysts, will be completely blinded to the treatment condition. Any adverse effects or body discomfort will be recorded after each rTMS treatment session. The aforementioned individuals will be unblinded to information about the treatment only when a participant experiences serious adverse events, and then an independent investigator will reveal the current treatment of this participant.

### Intervention

We will perform rTMS with the CCY-I magnetic field stimulator (Wuhan Yiruide Co., Ltd.), which can adjust the stimulation frequency from 0 to 100 Hz through a circular coil. Before the first treatment, single-pulse TMS will be administered to the primary motor cortex to elicit a discernible hand muscle response in at least three of five consecutive pulses to determine the resting motor threshold (RMT). The stimulation site of rTMS is the right DLPFC, which is located 5 cm anterior to the site of maximal stimulation of the first dorsal interossei in a parasagittal plane. To ensure better positioning of the TMS coil, we further use the scalp region used for F4 EEG electrode placement in the 10–20 International System (38, 39). The right DLPFC is known as one part of the socially related brain areas, plays a crucial role in the integration of goals and the reward system, has been found the weak responses in ASDs and selected for treating primary insomnia with good effects (23, 24). In addition, another study using 25% MT for the treatment of ASD found that it could alleviate sleep disturbances with little adverse effects (29). In this ongoing clinical trial, 1 Hz rTMS at 50% RMT (50 trains of 30 pulses, with a 3-s interval between each, leading to a sum of 1,500 pulses) will be applied over the right DLPFC. The treatment time will be determined according to the specific conditions of the subject, but the daily treatment time needs to be consistent (afternoon, the error cannot exceed 1 h). The operation process of the sham group will be the same as that of the rTMS treatment group, with no magnetic field passing through the skull. There will be no difference between the appearance and sound of the devices in the rTMS group and the sham group.

### Assessment

The assessments at baseline, after the 4-week treatment period, and the 4-week follow-up are summarized in Table 1.

**Table 1:** Summary of the assessments.

	**Randomization**		
**Assessments**	**Baseline**	**After 4 weeks of rTMS treatment**	**Follow-up at 4 weeks**
Informed consent	√		
ADI-R	√		
ADOS	√		
Home-PSG	√	√	√
CSHQ	√	√	√
CARS	√	√	√
ABC	√	√	√
SRS	√	√	√
RBS-R	√	√	√
Blood	√	√	√

rTMS, repetitive transcranial magnetic stimulation; ADI-R, Autism Diagnostic Interview-Revised; ADOS, Autism Diagnostic Observational Schedule; PSG, polysmongraphy; CSHQ, Children Sleep Habits Questionnaire; CARS, Childhood Autism Rating Scale; ABC, Aberrant Behavior Checklist; SRS, Social Responsiveness Scale; RBS-R, Repetitive Behavior Scale-Revised. Home-PSG will be measured in two consecutive nights. Sleep diary will be record for at least 7 consecutive days before treatment, during the treatment and follow-up period. Adverse events report will be filled every day after randomization.

#### The clinical and sleep assessment

Through the self-designed questionnaire, demographic information, including race, sex, age, weight, height, past and current illnesses and medications, family income, the parent's educational level, mother's pregnancy disease, and childbirth, will be obtained.

##### Sleep studies

The sleep diary will be sent to parents to record their child's sleeping habits, including the child's bedtime, the time at which the child left his or her bed in the morning, the time to falling asleep, the number of times the child woke up and fell back asleep, and the total sleep time, for at least 7 consecutive days before treatment. These are also recorded during treatment and follow-up.

The Children Sleep Habits Questionnaire (CSHQ) is a parent-reported questionnaire (40). It consists of 41 questions that can reflect the common sleep problems in a typical recent week at 8 different levels: bedtime resistance, sleep onset delay, sleep duration, sleep anxiety, night waking, parasomnias, sleep-disordered breathing, and daytime sleepiness. A total score higher than 41 indicates sleep disturbance. The scale has adequate internal consistency, acceptable test-retest reliability, and acceptable validity to be widely used to evaluate sleep problems among different ages of ASD patients.

To objectively assess the change in sleep parameters and overcome the first-night effect, we will bring portable equipment to the patient's home for two consecutive overnight polysomnography examinations. Sleep data will be collected and scored *via* Somté PSG (Compumedics, OR). Measures will include six electroencephalographic (EEG) leads (F3-M2, F4-M1, C3-M2, C4-M1, O1-M2, O2-M1), two electrooculographic leads (ROC-M2, LOC-M2), 2 x Chin electromyography (EMG), 2 x Limb EMG, airflow from the pressure transducer and thermistor, thoracic and abdominal inductive RIP band, position, finger pulse oximetry, and electrocardiogram. Recording techniques and estimation of sleep parameters will follow the American Academy of Sleep Medicine guidelines (V.2.3).

##### Clinical assessment and questionnaires

The four scales below will be used to assess the changes in clinical symptoms from different aspects (severity, aberrant/repetitive behavior, social responsiveness) at baseline, after treatment and at the one-month follow-up (Figure 1).

The Childhood Autism Rating Scale (CARS) will be used to diagnose ASD by clinicians (41). Based on the levels of social, emotional, adaptive, communicative, and cognitive aspects, the CARS can divide ASD patients into three different severity groups. ASD severity will be graded by CARS as none to minimal symptoms (15–30 points), mild to moderate symptoms (30–37 points), and severe symptoms (38–60 points). We will use this scale to assess the severity of changes at the above three time points.

The Aberrant Behavior Checklist (ABC) will be used to evaluate the main ASD symptoms, including irritability, lethargy/social withdrawal, stereotypic behavior, hyperactivity/noncompliance, and inappropriate speech (42). ABC in Chinese has satisfactory psychometric properties and has been widely used in the evaluation of treatment efficiency.

The Social Responsiveness Scale (SRS) is a parent rating scale that is used to measure the severity or quantities of social impairment in clinical samples, including ASD (43). It consists of 65 items including five theoretical subscale scores (labeled social awareness, social cognition, social communication, social motivation, and autistic mannerisms). The total raw score ranges from 0 to 195, with higher scores indicating increased social impairment. We will use this scale to assess changes in social responsiveness.

The Repetitive Behavior Scale-Revised (RBS-R) is used to measure the various repetitive behaviors among individuals with ASD (44). There are 43 items rated for severity on a 4-point Likert scale. The total raw score ranges from 0 to 172, and higher scores indicate serious repetitive behaviors.

#### Untargeted metabolomics analysis

We will collect whole blood using a heparin sodium anticoagulant tube and perform plasma separation as soon as possible. Briefly, the sample will be centrifuged at 3,000 rpm at 4°C for 10 min, the upper layer will be collected, and 0.2 mL/tube will be transferred into 2 mL centrifuge tubes. After the sample is labeled, it will be quick-frozen in liquid nitrogen for 15 min and stored at −80°C. Then, the sample will be incubated on ice for 5 min and centrifuged at 15,000 rpm for 5 min at 4°C. Some of the supernatant will be diluted to a final concentration containing 53% methanol with LC–MS grade water. The sample will be subsequently transferred to a fresh Eppendorf tube and then centrifuged at 15,000 g for 10 min at 4°C. Finally, the supernatant will be injected into the LC–MS/MS system for analysis. The raw data files generated by UHPLC–MS/MS will be processed using Compound Discoverer 3.1 (CD3.1, Thermo Fisher) to perform peak alignment, peak picking, and quantitation for each metabolite. These metabolites will be annotated using the KEGG database (http://www.genome.jp/kegg/), HMDB database (http://www.hmdb.ca/), and Lipidmaps database (http://www.lipidmaps.org/).

### Outcomes

The primary outcome of this study will be the difference in sleep onset latency between baseline and the end of treatment. Secondary outcomes will be differences in other parameters derived from polysomnography, the sleep diary, CSHQ, CARS, ABC, SRS, RBS-R, and metabolomics analysis.

#### Safety outcomes

Family members will also be asked to complete an adverse event report every day, and this report will include information about potential side effects, including headache, nausea, vomiting, and seizures. If the above symptoms occur during treatment, treatment will be immediately stopped to see if these symptoms subside. If status epilepticus occurs, the patient will be sent to the emergency room for symptomatic treatment. Safety outcomes and treatment-related side effects will be assessed and reported in the publication.

#### Follow-up

Four weeks after the last rTMS or sham treatment, parents and patients will be asked to return for a reevaluation of symptoms using the above scale. The CSHQ and home-PSG will be then used to assess sleep quality. In addition, whole blood samples will be collected again.

### Data management

A standardized case report form (CRF) was developed for the current study. After the clinical symptoms of the patients are evaluated at the specified time points, the data will be entered into the Excel database immediately. To ensure the accuracy of the data, another researcher will double-check.

## Statistical analysis

We will use SPSS software version 26.0 (IBM Corp, Armonk, New York, US) to perform data analysis. Demographic and baseline data will be analyzed with standard descriptive statistics. Normally and non-normally distributed data will be presented as means (SDs) and medians (quartiles), respectively. The comparison of basic data between the rTMS group and sham rTMS group will be performed using Student's *t*-test, the chi-squared test, or the nonparametric test according to the data type. To verify the efficacy of our intervention, the final analysis will be conducted according to the intent-to-treat (ITT) principle, and missing data will be replaced by means of the last observation carried forward rules. Changes in quantitative variables over time (evaluation of the therapeutic effect of rTMS on variations in sleep latency) will be analyzed using random-effect models allowing us to take into account repeated measurements. Time (baseline, post, and 1-month follow-up) and group (rTMS or sham) effects will be estimated and tested, along the interaction with the group. According to the same strategy, changes in other quantitative variables over time will be investigated. To further analyze the relationship between the change in the parameter of sleep and clinical symptoms, logistic regression, general linear correlation or regression will be taken into consideration between various types of outcomes. The number and proportions of participants with side effects, adverse events, and serious adverse events will also be reported. All statistical analyses will be two-sided, and statistical significance will be set at p<0.05. For untargeted metabolomics analysis, principal component analysis (PCA) and partial least squares discriminant analysis (PLS-DA) will be performed in metaX (a flexible and comprehensive software for processing metabolomics data). We will apply univariate analysis (*t*-test) to calculate the statistical significance (*P* value). Metabolites with VIP > 1 and *P* value < 0.05 and fold change (FC)≥ 2 or FC ≤ 0.5 will be considered to be differential metabolites.

## Discussion

This study protocol presents the design of an RCT investigating the therapeutic effects of rTMS over the right DLPFC for insomnia in ASD by using a combined approach of home-PSG, sleep diary and clinically relevant scores from participants and caregivers. The results of this study will provide preliminary data on whether rTMS can alleviate insomnia in patients with ASD.

Although the cause of sleep disturbances in children with ASD is not clear, it has been found that the hyperarousal state also exists in ASD with insomnia (45, 46). Apart from that, there are several other possible hypotheses to explain it. First, the underlying abnormal pathophysiological mechanism of ASD may be the direct cause of sleep disturbances (47). Abnormal neural connectivity in the brain plays a pivotal role in ASD. The DLPFC, an essential part of integrating distributed brain networks that support the operation of verbal/auditory and spatial information in the brain, is dysfunctional according to proton magnetic resonance spectroscopy data in ASD (48, 49). Furthermore, abnormal sleep-promoting transmitters such as melatonin and GABAergic circuits have been found in ASD children with sleep disturbances (50–52). Second, the important role of cortical excitation/inhibition balance has been confirmed in sleep-stage-specific regulation (53). Increased cortical excitability has been found in different types of insomnia patients (54). In ASD, studies have found that cortical inhibition decreases and that the balance is disrupted, a noted predictor of autistic phenotypes (55). Third, there is a bidirectional association between ASD and sleep disturbances. Sleep disturbances may be related to the clinical phenotype and core symptoms of ASD (6, 56). Fourth, sleep problems may be associated with psychiatric comorbidity and independent of ASD (47).

rTMS is a noninvasive technique that produces a magnetic field by passing a brief electric current and introduces excitatory or inhibitory cortical excitability in a small area of the brain below the magnetic coil by modulating different stimulus frequencies (>1 Hz or < 1 Hz) (57). Recently, rTMS has been used in ASD. Two double-blind, randomized trials found that high-frequency rTMS (5 Hz and 20 Hz) applied to the DLPFC or bilateral dorsal medial prefrontal cortex can improve anxiety and executive function performance in patients with ASD (33, 34). High-frequency rTMS (10 Hz) to the left DLPFC can also alleviate depressive symptoms among ASD patients with depression (58). Low-frequency rTMS can increase cardiac vagal control and reduce sympathetic arousal, alleviating behavioral problems post-TMS improvement (59, 60). One study adopted low-frequency rTMS in children with low-functioning autism and found that it can increase the peak alpha frequency in different brain regions and alleviate behavioral symptoms assessed by the ABC (61). Furthermore, meta-analyses and systematic reviews indicated that rTMS could help treat some dimensions of ASD, such as repetitive and stereotyped behavior, sociability, or some aspects of executive and cognitive functions (62, 63). One study adopted theta burst stimulation (TBS), a patterned repetitive transcranial magnetic stimulation (rTMS) protocol over the bilateral posterior superior temporal sulcus and proved that it had an immediate effect on parent-rate autistic symptoms in adults with ASD (64). For glutamate and GABAergic circuits that are abnormal in ASD, one study found that rTMS (1 Hz) treatment for 2 weeks effectively alleviated the acquired autistic-like symptoms in a rat model by regulating synaptic GABA transmission (65). Furthermore, in adults with ASD, one study found that rTMS to the DLPFC may modulate local glutamate levels (66).

Considering its potential role, we hypothesize that rTMS can alleviate insomnia symptoms in ASD. However, no study has explored the effect of rTMS on insomnia symptoms in objective ways (such as PSG) in ASD. Therefore, the results of this study may address an important knowledge gap and may provide evidence for noninvasive and nonpharmaceutical interventions to treat insomnia in ASD.

## Trial status

The described trial is ongoing, and recruitment commenced in July 2021. However, an overall slow rate of progress is due to COVID-19 restrictions. The data collection will continue until 30 participants completed all intervention sessions and the corresponding pre- and post-measurements. Follow-ups will be conducted 1 month after the stimulation sessions to investigate long-lasting effects.

## Ethics statement

The studies involving human participants were reviewed and approved by Ethics Committee on Biomedical Research, West China Hospital of Sichuan University. Written informed consent to participate in this study was provided by the participants' legal guardian/next of kin.

## Author contributions

JJ, LT, YZ, TL, and XT designed the trial. JJ, LT, and XT designed the stimulation model and defined the stimulation parameters. YZ and TL planned the data analysis. JJ is responsible for the recruitment of the participants and the data collection during the study and drafted the manuscript. YZ, LT, and XT edited and revised the paper. All authors approved the final manuscript.

## Funding

This work was supported by the Ministry of Science and Technology of the People's Republic of China (2021ZD0201900) and the National Natural Science Foundation of China (82120108002 and U21A20335).

## Conflict of interest

The authors declare that the research was conducted in the absence of any commercial or financial relationships that could be construed as a potential conflict of interest.

## Publisher's note

All claims expressed in this article are solely those of the authors and do not necessarily represent those of their affiliated organizations, or those of the publisher, the editors and the reviewers. Any product that may be evaluated in this article, or claim that may be made by its manufacturer, is not guaranteed or endorsed by the publisher.

## References

[1] Lord C Elsabbagh M Baird G Veenstra-Vanderweele J. Autism spectrum disorder. Lancet. (2018) 392:508–20. 10.1016/S0140-6736(18)31129-2 30078460 PMC7398158

[2] Lai MC Lombardo MV Baron-Cohen S. Autism. Lancet. (2014) 383:896–910. 10.1016/S0140-6736(13)61539-1 24074734

[3] Baio J Wiggins L Christensen DL Maenner MJ Daniels J Warren Z . Prevalence of autism spectrum disorder among children aged 8 years - autism and developmental disabilities monitoring network, 11 Sites, United States, 2014. MMWR Surveill Summ. (2018) 67:1–23. 10.15585/mmwr.ss6706a1 29701730 PMC5919599

[4] Reynolds AM Malow BA. Sleep and autism spectrum disorders. Pediatr Clin North Am. (2011) 58:685–98. 10.1016/j.pcl.2011.03.009 21600349

[5] Cohen S Conduit R Lockley SW Rajaratnam SM Cornish KM. The relationship between sleep and behavior in autism spectrum disorder (ASD): A review. J Neurodev Disord. (2014) 6:44. 10.1186/1866-1955-6-44 25530819 PMC4271434

[6] Mazzone L Postorino V Siracusano M Riccioni A Curatolo P. The relationship between sleep problems, neurobiological alterations, core symptoms of autism spectrum disorder, and psychiatric comorbidities. J Clin Med. (2018) 7:102. 10.3390/jcm7050102 29751511 PMC5977141

[7] Verhoeff ME Blanken L Kocevska D Mileva-Seitz VR Jaddoe V White T . The bidirectional association between sleep problems and autism spectrum disorder: a population-based cohort study. Mol Autism. (2018) 9:8. 10.1186/s13229-018-0194-8 29423134 PMC5791216

[8] Liu R Dong H Wang Y Lu X Li Y Xun G . Sleep problems of children with autism may independently affect parental quality of life. Child Psychiatry Hum Dev. (2021) 52:488–99. 10.1007/s10578-020-01035-z 32725386

[9] Esposito D Belli A Ferri R Bruni O. Sleeping without prescription: Management of sleep disorders in children with autism with non-pharmacological interventions and over-the-counter treatments. Brain Sci. (2020) 10:441. 10.3390/brainsci10070441 32664572 PMC7407189

[10] Williams BA Hirtz D Oskoui M Armstrong MJ Batra A Bridgemohan C . Practice guideline: Treatment for insomnia and disrupted sleep behavior in children and adolescents with autism spectrum disorder: Report of the guideline development, dissemination, and implementation subcommittee of the American Academy of Neurology. Neurology. (2020) 94:392–404. 10.1212/WNL.0000000000009033 32051244 PMC7238942

[11] McCrae CS Chan WS Curtis AF Deroche CB Munoz M Takamatsu S . Cognitive behavioral treatment of insomnia in school-aged children with autism spectrum disorder: a pilot feasibility study. Autism Res. (2020) 13:167–76. 10.1002/aur.2204 31566918

[12] Malow BA Byars K Johnson K Weiss S Bernal P Goldman SE . A practice pathway for the identification, evaluation, and management of insomnia in children and adolescents with autism spectrum disorders. Pediatrics. (2012) 130 Suppl 2:S106–24. 10.1542/peds.2012-0900I 23118242 PMC9923883

[13] Gringras P Nir T Breddy J Frydman-Marom A Findling RL. Efficacy and safety of pediatric prolonged-release melatonin for insomnia in children with autism spectrum disorder. J Am Acad Child Adolesc Psychiatry. (2017) 56:948–57. 10.1016/j.jaac.2017.09.414 29096777

[14] Mindell JA Emslie G Blumer J Genel M Glaze D Ivanenko A . Pharmacologic management of insomnia in children and adolescents: consensus statement. Pediatrics. (2006) 117:e1223–32. 10.1542/peds.2005-1693 16740821

[15] Ballester P Richdale AL Baker EK Peiró AM. Sleep in autism: a biomolecular approach to aetiology and treatment. Sleep Med Rev. (2020) 54:101357. 10.1016/j.smrv.2020.101357 32759030

[16] Lefaucheur JP Aleman A Baeken C Benninger DH Brunelin J Di Lazzaro V . Evidence-based guidelines on the therapeutic use of repetitive transcranial magnetic stimulation (rTMS): An update (2014-2018). Clin Neurophysiol. (2020) 131:474–528. 10.1016/j.clinph.2019.11.002 31901449

[17] McClintock SM Reti IM Carpenter LL McDonald WM Dubin M Taylor SF . Consensus recommendations for the clinical application of repetitive transcranial magnetic stimulation (rTMS) in the treatment of depression. J Clin Psychiatry. (2018) 79:16cs10905. 10.4088/JCP.16cs10905 28541649 PMC5846193

[18] Zewdie E Ciechanski P Kuo HC Giuffre A Kahl C King R . Safety and tolerability of transcranial magnetic and direct current stimulation in children: Prospective single center evidence from 35 million stimulations. Brain Stimul. (2020) 13:565–75. 10.1016/j.brs.2019.12.025 32289678

[19] Malone LA Sun LR. Transcranial magnetic stimulation for the treatment of pediatric neurological disorders. Curr Treat Options Neurol. (2019) 21:58. 10.1007/s11940-019-0600-3 31720969

[20] Oberman LM Enticott PG Casanova MF Rotenberg A Pascual-Leone A McCracken JT. Transcranial magnetic stimulation in autism spectrum disorder: challenges, promise, and roadmap for future research. Autism Res. (2016) 9:184–203. 10.1002/aur.1567 26536383 PMC4956084

[21] Huashuang Z Yang L Chensheng H Jing X Bo C Dongming Z . Prevalence of adverse effects associated with transcranial magnetic stimulation for autism spectrum disorder: a systematic review and meta-Analysis. Front Psychiatry. (2022) 13:875591. 10.3389/fpsyt.2022.875591 35677871 PMC9168239

[22] Nardone R Sebastianelli L Versace V Brigo F Golaszewski S Pucks-Faes E . Effects of repetitive transcranial magnetic stimulation in subjects with sleep disorders. Sleep Med. (2020) 71:113–21. 10.1016/j.sleep.2020.01.028 32173186

[23] Sun N He Y Wang Z Zou W Liu X. The effect of repetitive transcranial magnetic stimulation for insomnia: a systematic review and meta-analysis. Sleep Med. (2021) 77:226–37. 10.1016/j.sleep.2020.05.020 32830052

[24] Herrero BA Bellemare A Beetz G Vinet SA Martel MO Lavigne GJ . The effects of non-invasive brain stimulation on sleep disturbances among different neurological and neuropsychiatric conditions: a systematic review. Sleep Med Rev. (2021) 55:101381. 10.1016/j.smrv.2020.101381 32992227

[25] Feng J Zhang Q Zhang C Wen Z Zhou X. The effect of sequential bilateral low-frequency rTMS over dorsolateral prefrontal cortex on serum level of BDNF and GABA in patients with primary insomnia. Brain Behav. (2019) 9:e1206. 10.1002/brb3.1206 30609300 PMC6379591

[26] Jones BE. Arousal and sleep circuits. Neuropsychopharmacol. (2020) 45:6–20. 10.1038/s41386-019-0444-2 31216564 PMC6879642

[27] Gottesmann C. GABA mechanisms and sleep. Neuroscience. (2002) 111:231–9. 10.1016/S0306-4522(02)00034-9 11983310

[28] Furihata R Saitoh K Otsuki R Murata S Suzuki M Jike M . Association between reduced serum BDNF levels and insomnia with short sleep duration among female hospital nurses. Sleep Med. (2020) 68:167–72. 10.1016/j.sleep.2019.12.011 32044553

[29] Gao L Wang C Song XR Tian L Qu ZY Han Y . The sensory abnormality mediated partially the efficacy of repetitive transcranial magnetic stimulation on treating comorbid sleep disorder in autism spectrum disorder children. Front Psychiatry. (2021) 12:820598. 10.3389/fpsyt.2021.820598 35140641 PMC8818693

[30] Sanchez-Escandon O Arana-Lechuga Y Teran-Perez G Ruiz-Chow A Gonzalez-Robles R Shkurovich-Bialik P . Effect of low-frequency repetitive transcranial magnetic stimulation on sleep pattern and quality of life in patients with focal epilepsy. Sleep Med. (2016) 20:37–40. 10.1016/j.sleep.2015.11.022 27318224

[31] Jiang CG Zhang T Yue FG Yi ML Gao D. Efficacy of repetitive transcranial magnetic stimulation in the treatment of patients with chronic primary insomnia. Cell Biochem Biophys. (2013) 67:169–73. 10.1007/s12013-013-9529-4 23797608

[32] Arazi A Meiri G Danan D Michaelovski A Flusser H Menashe I . Reduced sleep pressure in young children with autism. Sleep. (2020) 43:zsz309. 10.1093/sleep/zsz309 31848619

[33] Ameis SH Blumberger DM Croarkin PE Mabbott DJ Lai MC Desarkar P . Treatment of executive function deficits in autism spectrum disorder with repetitive transcranial magnetic stimulation: a double-blind, sham-controlled, pilot trial. Brain Stimul. (2020) 13:539–47. 10.1016/j.brs.2020.01.007 32289673 PMC8129776

[34] Enticott PG Fitzgibbon BM Kennedy HA Arnold SL Elliot D Peachey A . A double-blind, randomized trial of deep repetitive transcranial magnetic stimulation (rTMS) for autism spectrum disorder. Brain Stimul. (2014) 7:206–11. 10.1016/j.brs.2013.10.004 24280031

[35] Lord C Risi S Lambrecht L Cook EJ Leventhal BL DiLavore PC . The autism diagnostic observation schedule-generic: a standard measure of social and communication deficits associated with the spectrum of autism. J Autism Dev Disord. (2000) 30:205–23. 10.1037/t17256-000 11055457

[36] Rutter M Le Couteur A Lord C. The Autism Diagnostic Interview-revised (ADI-R). Los Angeles, CA: Western Psychological Services. (2003).

[37] Sateia MJ. International classification of sleep disorders-third edition: highlights and modifications. Chest. (2014) 146:1387–94. 10.1378/chest.14-0970 25367475

[38] Yang Y Wang H Xue Q Huang Z Wang Y. High-frequency repetitive transcranial magnetic stimulation applied to the parietal cortex for low-functioning children with autism spectrum disorder: a case series. Front Psychiatry. (2019) 10:293. 10.3389/fpsyt.2019.00293 31143132 PMC6520602

[39] Herwig U Satrapi P Schonfeldt-Lecuona C. Using the international 10-20 EEG system for positioning of transcranial magnetic stimulation. Brain Topogr. (2003) 16:95–9. 10.1023/B:BRAT.0000006333.93597.9d 14977202

[40] Tan TX Wang Y Cheah C Wang GH. Reliability and construct validity of the Children's Sleep Habits Questionnaire in Chinese kindergartners. Sleep Health. (2018) 4:104–9. 10.1016/j.sleh.2017.10.008 29332670

[41] Schopler E Reichler RJ DeVellis RF Daly K. Toward objective classification of childhood autism: childhood Autism Rating Scale (CARS). J Autism Dev Disord. (1980) 10:91–103. 10.1007/BF02408436 6927682

[42] Aman MG Singh NN Stewart AW Field CJ. The aberrant behavior checklist: a behavior rating scale for the assessment of treatment effects. Am J Ment Defic. (1985) 89:485–91. 10.1037/t10453-000 3993694

[43] Constantino JN Davis SA Todd RD Schindler MK Gross MM Brophy SL . Validation of a brief quantitative measure of autistic traits: comparison of the social responsiveness scale with the autism diagnostic interview-revised. J Autism Dev Disord. (2003) 33:427–33. 10.1023/A:1025014929212 12959421

[44] He H Ye N Yi L Yang C. Validating the Repetitive Behavior Scale-Revised for children in China aged 3 to 8 with autism spectrum disorder. J Autism Dev Disord. (2019) 49:4941–56. 10.1007/s10803-019-04210-x 31485814

[45] Baker EK Richdale AL Hazi A Prendergast LA. Assessing a hyperarousal hypothesis of insomnia in adults with autism spectrum disorder. Autism Res. (2019) 12:897–910. 10.1002/aur.2094 30896090

[46] Riemann D Spiegelhalder K Feige B Voderholzer U Berger M Perlis M . The hyperarousal model of insomnia: a review of the concept and its evidence. Sleep Med Rev. (2010) 14:19–31. 10.1016/j.smrv.2009.04.002 19481481

[47] Missig G McDougle CJ Carlezon WJ. Sleep as a translationally-relevant endpoint in studies of autism spectrum disorder (ASD). Neuropsychopharmacol. (2020) 45:90–103. 10.1038/s41386-019-0409-5 31060044 PMC6879602

[48] Solomon M Yoon JH Ragland JD Niendam TA Lesh TA Fairbrother W . The development of the neural substrates of cognitive control in adolescents with autism spectrum disorders. Biol Psychiatry. (2014) 76:412–21. 10.1016/j.biopsych.2013.08.036 24209777 PMC3999330

[49] Herrington JD Riley ME Grupe DW Schultz RT. Successful face recognition is associated with increased prefrontal cortex activation in autism spectrum disorder. J Autism Dev Disord. (2015) 45:902–10. 10.1007/s10803-014-2233-4 25234479 PMC4366341

[50] Miyoshi G Ueta Y Natsubori A Hiraga K Osaki H Yagasaki Y . FoxG1 regulates the formation of cortical GABAergic circuit during an early postnatal critical period resulting in autism spectrum disorder-like phenotypes. Nat Commun. (2021) 12:3773. 10.1038/s41467-021-23987-z 34145239 PMC8213811

[51] Wood ET Cummings KK Jung J Patterson G Okada N Guo J . Sensory over-responsivity is related to GABAergic inhibition in thalamocortical circuits. Transl Psychiatry. (2021) 11:39. 10.1038/s41398-020-01154-0 33436538 PMC7804323

[52] Paulsen B Velasco S Kedaigle AJ Pigoni M Quadrato G Deo AJ . Autism genes converge on asynchronous development of shared neuron classes. Nature. (2022) 602:268–73. 10.1038/s41586-021-04358-6 35110736 PMC8852827

[53] Niethard N Hasegawa M Itokazu T Oyanedel CN Born J Sato TR. Sleep-stage-specific regulation of cortical excitation and inhibition. Curr Biol. (2016) 26:2739–49. 10.1016/j.cub.2016.08.035 27693142

[54] Lanza G Cantone M Lanuzza B Pennisi M Bella R Pennisi G . Distinctive patterns of cortical excitability to transcranial magnetic stimulation in obstructive sleep apnea syndrome, restless legs syndrome, insomnia, and sleep deprivation. Sleep Med Rev. (2015) 19:39–50. 10.1016/j.smrv.2014.04.001 24849846

[55] Oliveira B Mitjans M Nitsche MA Kuo MF Ehrenreich H. Excitation-inhibition dysbalance as predictor of autistic phenotypes. J Psychiatr Res. (2018) 104:96–9. 10.1016/j.jpsychires.2018.06.004 30015265

[56] Schreck KA Richdale AL. Sleep problems, behavior, and psychopathology in autism: Inter-relationships across the lifespan. Curr Opin Psychol. (2020) 34:105–11. 10.1016/j.copsyc.2019.12.003 31918238

[57] Perera T George MS Grammer G Janicak PG Pascual-Leone A Wirecki TS. The clinical TMS society consensus review and treatment recommendations for TMS therapy for major depressive disorder. Brain Stimul. (2016) 9:336–46. 10.1016/j.brs.2016.03.010 27090022 PMC5612370

[58] Gwynette MF Lowe DW Henneberry EA Sahlem GL Wiley MG Alsarraf H . Treatment of adults with autism and major depressive disorder using transcranial magnetic stimulation: an open label pilot study. Autism Res. (2020) 13:346–51. 10.1002/aur.2266 31944611 PMC10150802

[59] Sokhadze EM El-Baz A Baruth J Mathai G Sears L Casanova MF. Effects of low frequency repetitive transcranial magnetic stimulation (rTMS) on gamma frequency oscillations and Event-Related potentials during processing of illusory figures in autism. J Autism Dev Disord. (2009) 39:619–34. 10.1007/s10803-008-0662-7 19030976

[60] Casanova MF Hensley MK Sokhadze EM El-Baz AS Wang Y Li X . Effects of weekly low-frequency rTMS on autonomic measures in children with autism spectrum disorder. Front Hum Neurosci. (2014) 8:851. 10.3389/fnhum.2014.00851 25374530 PMC4204613

[61] Kang JN Song JJ Casanova MF Sokhadze EM Li XL. Effects of repetitive transcranial magnetic stimulation on children with low-function autism. Cns Neurosci Ther. (2019) 25:1254–61. 10.1111/cns.13150 31228356 PMC6834922

[62] Khaleghi A Zarafshan H Vand SR Mohammadi MR. Effects of non-invasive neurostimulation on autism spectrum disorder: a systematic review. Clin Psychopharmacol Neurosci. (2020) 18:527–52. 10.9758/cpn.2020.18.4.527 33124586 PMC7609207

[63] Barahona-Corrêa JB Velosa A Chainho A Lopes R Oliveira-Maia AJ. Repetitive transcranial magnetic stimulation for treatment of autism spectrum disorder: a systematic review and meta-analysis. Front Integr Neurosci. (2018) 12:27. 10.3389/fnint.2018.00027 30038561 PMC6046620

[64] Ni HC Lin HY Chen YL Hung J Wu CT Wu YY . 5-Day multi-session intermittent theta burst stimulation over bilateral posterior superior temporal sulci in adults with autism-a pilot study. Biomed J. (2021). 10.1016/j.bj.2021.07.008 34358713 PMC9486126

[65] Tan T Wang W Xu H Huang Z Wang YT Dong Z. Low-frequency rTMS ameliorates autistic-like behaviors in rats induced by neonatal isolation through regulating the synaptic GABA transmission. Front Cell Neurosci. (2018) 12:46. 10.3389/fncel.2018.00046 29541022 PMC5835518

[66] Moxon-Emre I Daskalakis ZJ Blumberger DM Croarkin PE Lyon RE Forde NJ . Modulation of dorsolateral prefrontal cortex glutamate/glutamine levels following repetitive transcranial magnetic stimulation in young adults with autism. Front Neurosci. (2021) 15:711542. 10.3389/fnins.2021.711542 34690671 PMC8527173

